# The Impact of Spaceflight and Microgravity on the Human Islet-1+ Cardiovascular Progenitor Cell Transcriptome

**DOI:** 10.3390/ijms22073577

**Published:** 2021-03-30

**Authors:** Victor Camberos, Jonathan Baio, Ana Mandujano, Aida F. Martinez, Leonard Bailey, Nahidh Hasaniya, Mary Kearns-Jonker

**Affiliations:** 1 Department of Pathology and Human Anatomy, Loma Linda University School of Medicine, Loma Linda, CA 92350, USA; vcamberos@students.llu.edu (V.C.); jonbaio13@gmail.com (J.B.); anamandu96@gmail.com (A.M.); afmartinez3@gmail.com (A.F.M.); 2 Department of Cardiovascular and Thoracic Surgery, Loma Linda University School of Medicine, Loma Linda, CA 92350, USA; llbailey@llu.edu (L.B.); nhasaniya@yahoo.com (N.H.)

**Keywords:** transcriptomics, spaceflight, microgravity, stemness, proliferation, miRNA, cardiovascular

## Abstract

Understanding the transcriptomic impact of microgravity and the spaceflight environment is relevant for future missions in space and microgravity-based applications designed to benefit life on Earth. Here, we investigated the transcriptome of adult and neonatal cardiovascular progenitors following culture aboard the International Space Station for 30 days and compared it to the transcriptome of clonally identical cells cultured on Earth. Cardiovascular progenitors acquire a gene expression profile representative of an early-stage, dedifferentiated, stem-like state, regardless of age. Signaling pathways that support cell proliferation and survival were induced by spaceflight along with transcripts related to cell cycle re-entry, cardiovascular development, and oxidative stress. These findings contribute new insight into the multifaceted influence of reduced gravitational environments.

## 1. Introduction

The effects of spaceflight and microgravity on humans are a continuously developing area of interest. Investigating the gene expression profile of cells derived from astronauts working on the International Space Station (ISS) or from cells cultured in space contributes critical knowledge towards understanding the impact of the spaceflight environment on human cells. Emerging evidence has identified important distinguishing differences following long- and short-term microgravity exposure [1,2]. While long-term effects are currently under investigation, short-term exposure to spaceflight may have beneficial applications here on Earth.

Reduced gravitational force exerted while on the ISS or simulated on Earth influences cell behavior, morphology, and mechanical stress [3,4,5] depending on cell type, differentiation stage, or exposure time [6,7]. Transcriptomic analysis defining the effects of spaceflight on both stem and fully differentiated cells provides a comprehensive and effective strategy for identifying how pathways and biological processes are affected by the spaceflight environment. One of the most intricate and elaborate transcriptomic studies regarding the effects of long-term spaceflight on the human body is the NASA Twin Study. In this study, the transcriptome of one twin brother was compared to the transcriptome of the other twin brother who had spent an entire year in space onboard the ISS [8]. Key findings reported in this study include increased expression of inflammatory markers, T cell differentiation transcripts, and cytokines associated with cell growth and proliferation in response to spaceflight. Recent findings from short-term microgravity experiments are beginning to reveal interesting changes in cell behavior during the acclimation to reduced gravity. Human induced pluripotent stem cell (iPSC)-derived cardiomyocytes cultured aboard the ISS for 5.5 weeks, for example, demonstrate a significant elevation of gene transcripts related to calcium signaling and hypertrophy [9]. Interestingly, these transcripts returned towards baseline expression after being cultured further upon returning to Earth. Several types of stem-like cells found within amphibians presented with an increase in proliferation and dedifferentiation markers as well as enhanced regenerative capacity following exposure to short-term spaceflight [10]. Similarly, we have previously shown that short-term simulated microgravity exposure as well as culture aboard the ISS is capable of inducing stemness and dedifferentiation transcripts in cardiovascular progenitor cells (CPCs) [11,12].

While the effects of spaceflight may differ significantly across models, investigating the impact of long-term space travel on distinct cardiovascular cell types will provide insight necessary to guide future space exploration. Meanwhile, the potential of short-term microgravity exposure for cardiovascular stem cell expansion and age-related disease modeling needs to be investigated for future application. Transcriptomic information will provide an important step in exploring these possibilities.

Here, our objective is to investigate the effects of the spaceflight environment on the total transcriptomic profile of human adult and neonatal (neo) cardiovascular progenitors cultured aboard the ISS for 30 days. Specifically, the following study focuses on the effects of reduced gravity experienced on the ISS with regard to cell stemness and supporting signaling pathways. We seek to understand the molecular mechanism regulating the cellular response to spaceflight.

## 2. Results

### 2.1. KEGG Analysis of Induced Gene Transcripts Reveals Broad Responses to Spaceflight

**Figure 1:**
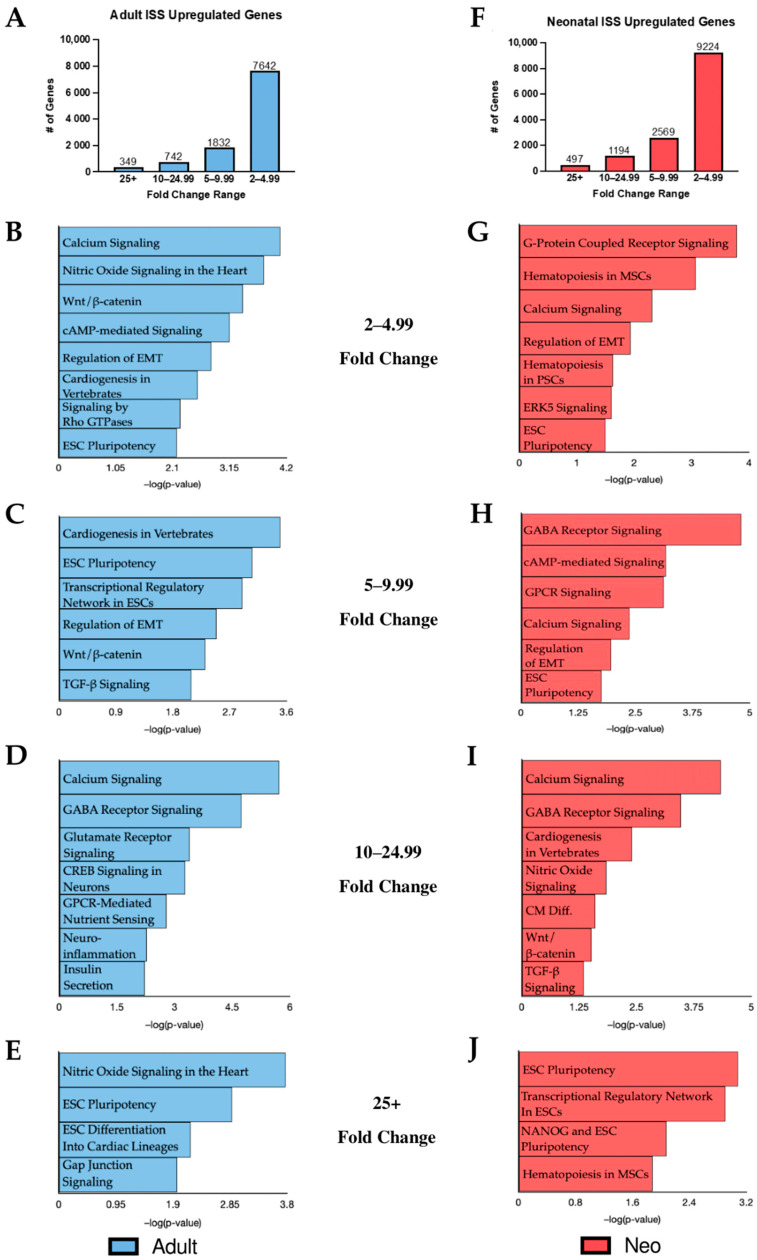
Induced transcripts from flown adult (left) and neonatal (right) cardiovascular progenitor cells were separated by fold change. (**A**) The distribution of upregulated transcripts in each fold change category is shown for flown adult CPCs. (**B**) KEGG analysis of transcripts with a fold change of 2–4.99, (**C**) 4–9.99, (**D**) 10–24.99, and (**E**) 25+ in flown adult CPCs. (**F**) The distribution of upregulated transcripts in each fold change category is shown for flown neonatal CPCs. (**G**) KEGG analysis of transcripts with a fold change of 2–4.99, (**H**) 4–9.99, (**I**) 10–24.99, and (**J**) 25+ in flown neonatal CPCs.

In order to deepen our understanding of the effects of spaceflight on cells in vitro, RNA was collected from CPCs following 30 days of culture aboard the ISS. Using RNA sequencing, the gene expression profile of flown adult and neonatal cells was compared against clonally identical cells cultured under normal gravity conditions. A fold change cutoff of 2.0+ was applied to the data as a means of focusing our attention on statistically significant gene expression changes. Compared to their respective ground controls, the adult population cultured in space displayed 10,565 upregulated transcripts while 13,484 transcripts were induced in the neonatal CPCs following culture on the ISS. Transcripts in the adult and neonatal populations were grouped according to fold change (Figure 1A,F). Upregulated gene transcripts with fold changes of 2–4.99, 5–9.99, 10–24.99, and 25+ were individually uploaded into Qiagen’s Ingenuity Pathway Analysis (IPA) software to identify canonical pathways induced by culture aboard the ISS (Figure 1B–E, adult; Figure 1G–J, neo). This approach highlighted both age-dependent and communal effects of spaceflight culture.

Several unique age-dependent effects of spaceflight were identified in this study when CPCs were compared against their respective ground controls. Signaling by Rho family GTPases appeared in the adult KEGG analysis but was not significantly induced in the neonates (Figure 1B). Rho GTPases play a role in cell migration and actin cytoskeleton remodeling [13]. Gap junction signaling, which is important for cell communication and normal heart contractility, was also uniquely significant in flown adult CPCs (Figure 1E) [14]. Recently, Gap junctions have been found to have the potential to modulate differentiation into cardiac lineages and enhance regenerative capacity [15]. G protein-coupled receptor (GPCR) signaling was uniquely impacted in flown neonatal CPCs and was the most significant category induced within the 2–4.99-fold change range, the range with the highest number of induced transcripts (Figure 1G). The role of GPCRs in promoting cell proliferation and self-renewal has been well established [16]. GPCRs are also abundant in the cardiovascular system and have significant roles in cell migration, differentiation, and cardiac contractility [17]. Additionally, ERK5 signaling was impacted by spaceflight in an age-dependent matter, significant only in the neonatal group. ERK5 signaling is a known regulator of cell cycle progression and proliferation and its induction has been associated with increased pluripotent potential [18,19].

Given the age-related differences observed following culture aboard the ISS, we briefly compared the transcriptome of the flown neonatal CPCs to that of the adults. KEGG analysis of the top 100 differentially expressed transcripts identified when comparing the flown neonatal CPCs to the flown adult CPCs revealed significant alterations in cytokine–cytokine receptor interaction as well as activation of the mitogen-activated protein kinase (MAPK) signaling pathway. Within the top 100 significantly altered transcripts group, the transcript with the highest fold change in the flown neonatal versus the flown adult dataset was the chemokine CXCR4 (875.04-fold) which is a key component of cytokine–cytokine receptor interactions. CXCR4 is a regulator of cell migration and pluripotency and is highly expressed in the regenerative zebrafish model [20]. Interestingly, there is a positive correlation between CXCR4 expression and MAPK signaling [21]. Interplay between MAPK and ERK5 signaling is well established. The finding that MAPK signaling is enhanced in flown neonatal CPCs relative to flown adult CPCs is consistent with activated ERK5 signaling in neonates relative to ground controls.

Our data suggest a general effect of spaceflight, regardless of age, on certain pathways and cellular processes in human CPCs. First, cardiac commitment markers MESP1 and NKX2-5 were induced in flown CPCs of both age groups (MESP1: 39.68- and 29.32-fold change in adult and neonatal CPCs, respectively; NKX2-5: 44.78- and 15.05-fold change in adult and neonatal CPCs, respectively). IPA analysis revealed several shared categories in the adult and neonatal CPC groups such as “Wnt/β-catenin Signaling”, “Regulation of EMT”, “Calcium Signaling”, and “Human ESC Pluripotency”. Wnt/β-catenin signaling plays a significant role in many developmentally necessary cellular processes such as differentiation, lineage commitment, survival, and cell proliferation [22]. Epithelial–mesenchymal transition (EMT) regulates cell differentiation during the developmental stages of life [23]. Calcium signaling is a key regulator of cell proliferation and plays a role in cell differentiation as well [24]. Pluripotency is a defining characteristic of stem cells and refers to a cell’s ability to self-renew and differentiate into other cell lineages. The presence of the human embryonic stem cell (ESC) pluripotency category in both the adult and neonatal groups may be an indication of increased stemness in response to spaceflight.

### 2.2. Spaceflight Induces Stemness

**Figure 2:**
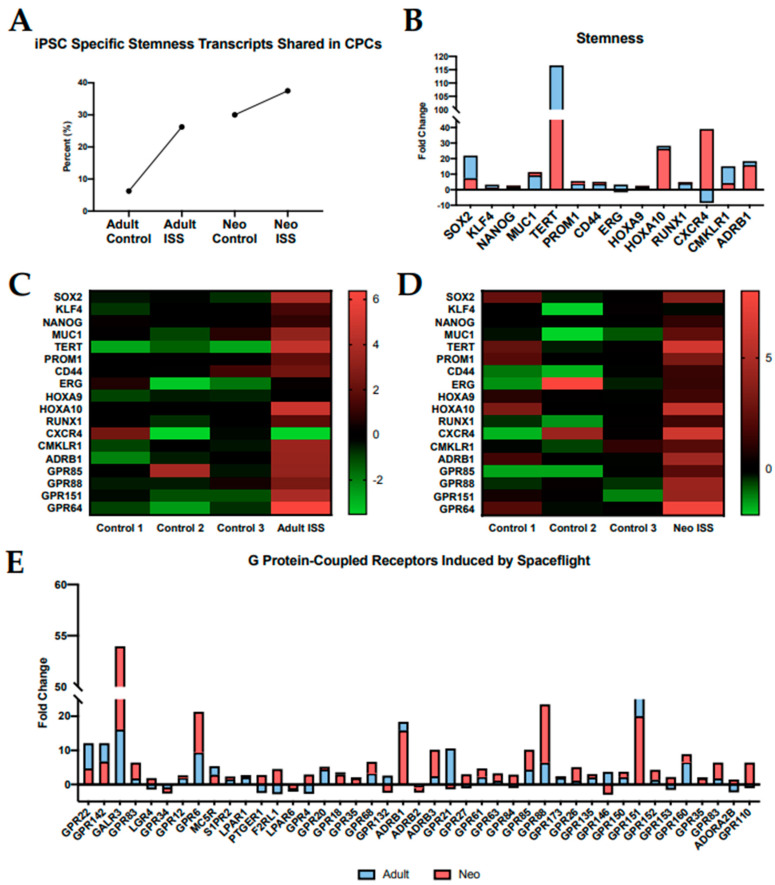
Spaceflight induces transcripts related to stemness. (**A**) StemChecker shows an increase in overlapping iPSC stemness markers in flown CPCs. (**B**) Other established stemness markers were impacted by spaceflight. (**C**) Differential gene expression in flown samples and ground controls in adult CPCs and (**D**) neonatal CPCs. (**E**) GPCR signaling was induced in CPCs cultured aboard the ISS. Individual fold changes for adult and neonatal groups are presented superimposed for comparison.

Stemness applies to cells which retain the ability to readily differentiate into downstream lineages and proliferate rapidly or self-renew. Given the results in Figure 1 regarding human ESC pluripotency, we investigated the impact of spaceflight on stemness in both neonatal and adult CPCs. In order to do this, we uploaded our list of significantly upregulated transcripts into StemChecker, a powerful online tool comprised of genes associated with stem cell function and characteristics [25]. StemChecker develops stemness signatures based on the most current literature regarding the most critically relevant genes that define stem cells and allows investigators to identify stemness overlap in their own uploaded datasets. Results from StemChecker demonstrated that the stemness signature of adult and neonatal cells was more comparable to that of iPSCs following spaceflight, potentially indicating an increase in stemness (Figure 2A). Given the proliferative nature of neonatal CPCs, the percent difference between flown and ground stemness signatures was less dramatic than in adults. Interestingly, after culture aboard the ISS, the percentage of overlapping stemness genes with iPSCs in adult CPCs is comparable to neonatal CPC ground controls. This finding is important considering the regenerative nature of neonatal CPCs and reveals the potential of a spaceflight environment to reprogram the gene expression profile of adult CPCs to be more similar to neonatal cells.

StemChecker analysis suggests that culture aboard the ISS for 30 days impacts stemness in our samples. In order to validate this, we next assessed the expression of specific genes related to stem cell maintenance (Figure 2B). Individual fold changes of stemness genes in flown adult and neonatal CPCs relative to their respective ground controls are shown superimposed in Figure 2B. A comprehensive list of fold changes that correspond to the genes presented here is openly accessible in FigShare. Adult and neonatal CPCs presented with elevated transcripts for Sox2 (21.92- and 7.39-fold change, respectively) and Klf4 (3.25- and 1.32-fold change, respectively) after being cultured aboard the ISS for 30 days. These genes are members of the Yamanaka family of transcription factors which are essential for reprogramming cells into iPSCs [26]. The induction of Sox2 and Klf4 is indicative of cellular reprogramming towards a more stem-like state. Interestingly, the induction of these gene transcripts was more pronounced in the adult CPCs. Heatmaps showing the change in expression for each individual gene in both the adult and neonatal groups are shown in Figure 2C,D, respectively. To further assess stemness in our flown samples, we investigated GPCR signaling which is known to regulate stem cell maintenance and pluripotency, as well as cell survival [27]. Fold changes for stemness-specific GPCRs that were altered in adult and neonatal CPCs cultured aboard the ISS relative to their respective ground controls are shown in Figure 2E. Activation of these GPCRs in response to spaceflight is suggestive of stem cell maintenance and proliferative capacity. While both age groups show GPCR induction to some extent, the expression levels are considerably higher in neonates compared to adults. This finding is consistent with the KEGG analysis in Figure 1 and is a possible indication of enhanced stemness in the neonatal CPCs when compared to their adult counterparts.

### 2.3. Culture Aboard the ISS for 30 Days Induced Transcripts with Dual Roles in Stemness and Senescence

**Figure 3:**
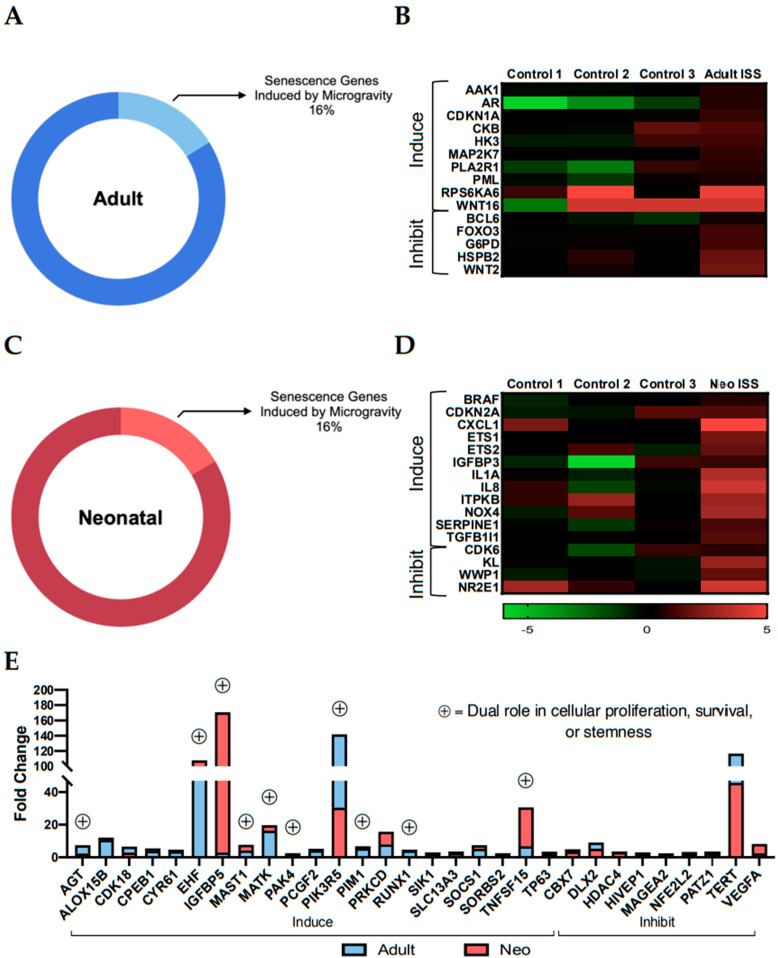
Spaceflight induced senescence genes that also play a role in stemness. (**A**) Adult CPCs induced 16% of senescence genes found in the CellAge database. (**B**) Ten senescence-inducing genes and five inhibiting genes were unique to the adult CPCs. (**C**) Neonatal CPCs induced 16% of CellAge senescence genes. (**D**) Sixteen genes were unique to neonatal CPCs: 12 inducing, 4 inhibiting. (**E**) Senescence-inducing genes that were shared between both age groups have dual roles in cell survival and proliferation.

Given the growing concern surrounding the effect of spaceflight on aging, we investigated the impact of the ISS environment on genes associated with senescence. Cellular senescence refers to the decline in proliferation or cellular growth and is a major component of the aging process [21]. We compared our list of induced transcripts to a list of senescence genes curated by CellAge, a part of the Human Ageing Genomic Resources (HAGR) database. The CellAge database consists of 279 genes that either induce (153), inhibit (121), or non-specifically affect (5) cellular senescence [22]. In the flown adult CPC transcriptome, 45 (16%) of the 279 senescence-associated genes were induced (Figure 3A). Of the 45 matched genes, 15 were unique to the adult population: 10 senescence-inducing and 5 inhibitory (Figure 3B). Similarly, neonates presented with an induction in 46 (16%) of the 279 senescence-associated genes post-flight (Figure 3C). Sixteen of the 46 genes were unique to the neonatal CPC transcriptome with 12 inducing genes and 4 inhibitory (Figure 3D). The spaceflight environment also induced 21 transcripts that correlate with the induction of senescence and 9 that inhibit senescence in human CPCs, irrespective of age (Figure 3E). While the senescence-inducing transcripts were more prevalent than the inhibitors in our data, many of them have dual roles in cell proliferation and stemness.

Senescence-associated stemness is a recently developed term that refers to the increase in stem-like characteristics in cells that had previously been considered senescent [28]. The shift in cell behavior is epigenetically driven and involves the activation of genes and signaling pathways that aid the bypassing of cell cycle arrest. In our data, for example, several of the senescence-associated genes that were most highly induced are also important in promoting cell survival or other stem cell features, as shown in Figure 3E (genes with symbol). Insulin like growth factor binding protein 5 (IGFBP5) expression is induced during senescence and is responsible for activating tumor suppressor p53, a key mediator of cell cycle arrest [29]. IGFBP5 was induced by spaceflight in adult and neonatal CPCs (2.97- and 170.77-fold change, respectively); meanwhile, p53 expression remained unchanged (1.52- and −1.39-fold change, respectively). An alternative downstream target of IGFBP5, integrin linked kinase (ILK), contributes to increased survival [30] plus enhanced proliferation, and AKT signaling activity [31]. Following culture aboard the ISS, ILK was induced in adult and neonatal CPCs by 8.45- and 7.73-fold change, respectively. ETS homologous factor (EHF) is considered a senescence-inducing gene based on its role in activating the senescence marker p16 (CDKN2A) which inhibits cell cycle progression when highly expressed [32]. EHF is highly induced in both adult and neonatal CPCs in response to spaceflight (100.0- and 107.81-fold change, respectively), yet CDKN2A is not (1.23- and 2.52-fold change, respectively). Interestingly, EHF is also known to play a role in stem cell maintenance and proliferation [33,34]. The induction of senescence-associated transcripts following culture aboard the ISS is not sufficient to claim that increased aging is occurring in CPCs, as many of the genes have dual functions in stemness processes. For this reason, it was necessary to investigate the impact of spaceflight on specific signaling pathways related to survival and proliferation.

### 2.4. Spaceflight Alters the Expression of Specific Pathways Related to Proliferation and Survival

**Figure 4:**
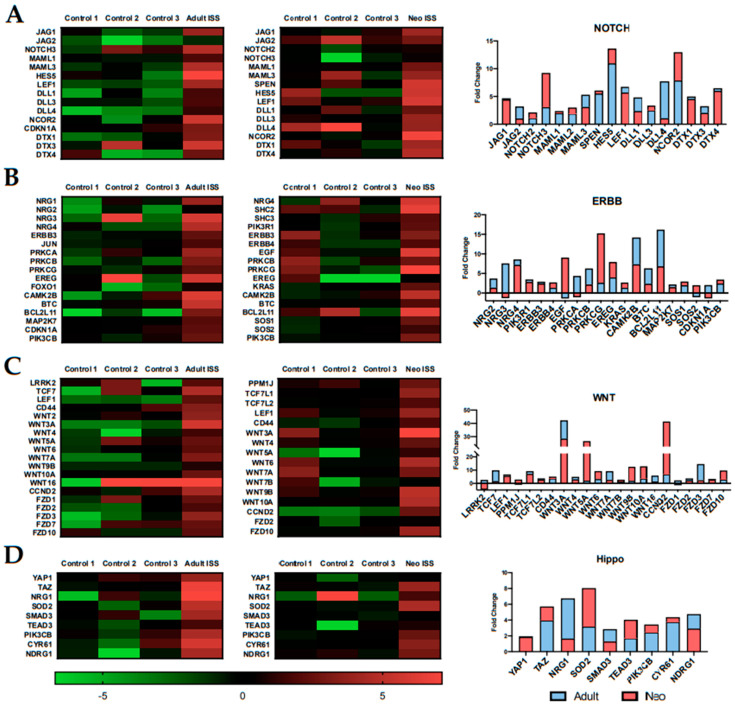
Pathways related to survival and proliferation were positively induced by spaceflight. Representative pathways include (**A**) Notch, (**B**) ERBB, (**C**) Wnt, and (**D**) Hippo.

We have previously reported that culturing CPCs aboard the ISS induces proliferation [35,36]. Here, we investigated the effects of spaceflight on specific survival and proliferation pathways. The Notch pathway is associated with cardiomyocyte proliferation and reduced fibrosis [37]. JAG1, a key component of the Notch pathway, along with other associated transcripts, was induced after 30 days of spaceflight (Figure 4A). ERBB signaling is necessary for functional cardiovascular development [38]. In spaceflight-cultured CPCs, members of the neuregulin (NRG) family and ERBB receptors were upregulated along with other transcripts related to ERBB signaling (Figure 4B). Transcriptomic analysis in the current study validated previous findings from our lab pertaining to the Wnt signaling pathway using focused pathway arrays [35]. Members of the Wnt family, specifically Wnt3a and Wnt5a, were induced in both adult and neonatal CPCs after 30 days of spaceflight (Figure 4C). Components of the Hippo pathway have been shown to regulate Notch [39], ERBB [40], and Wnt [41] signaling pathways and were induced by the spaceflight environment (Figure 4D).

### 2.5. Spaceflight Alters the Expression of Genes Related to Specific Biological Processes

**Figure 5:**
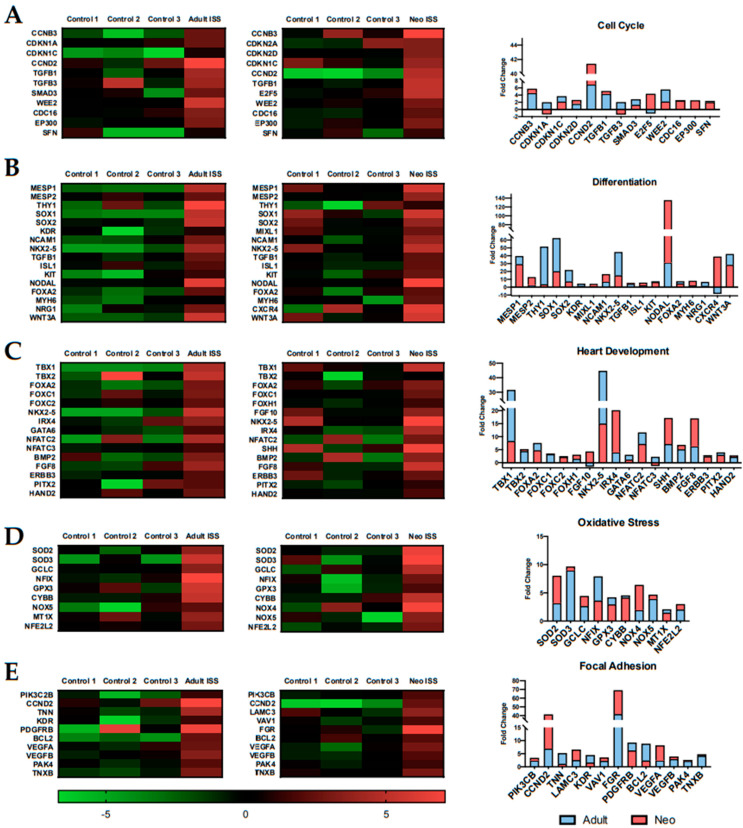
Gene transcripts associated with repair mechanisms were induced in flown CPCs compared to ground controls. Reparative processes that were induced by spaceflight include (**A**) cell cycle, (**B**) differentiation, (**C**) heart development, (**D**) oxidative stress, and (**E**) focal adhesion.

Genes related to several biologic processes were differentially expressed after 30 days of spaceflight. For example, key transcripts involved in cell cycle progression, cell differentiation, heart development, oxidative stress, and focal adhesion were induced in both adult and neonatal CPCs following culture aboard the ISS (Figure 5). Cyclins and cyclin-dependent kinases, which are involved in cell cycle progression and the promotion of proliferation, were significantly induced [42] (Figure 5A). Additionally, SMAD3 which regulates CCND2 and other cell cycle genes was upregulated [43]. Genes involved in stem cell differentiation were significantly induced in our flown samples (Figure 5B). The most highly induced gene, Nodal, plays a critical role in cell proliferation and is necessary for mesoderm and endoderm differentiation [44]. The ability to readily differentiate into downstream cardiovascular lineages is important in the context of regenerative medicine. Differentiation is regulated by Nkx2-5, which is a gene related to heart development [45,46]. In our spaceflight samples, Nkx2-5 transcripts were induced relative to ground controls (Figure 5C). Oxidative stress genes are important in a repair model of myocardial infarction to reduce the negative effects of reactive oxygen species that may arise following myocardial infarction [47]. Superoxide dismutase 2 (SOD2), which protects the cardiovascular system from reactive oxygen species and activates the Akt signaling pathway [48], was significantly induced in our spaceflight samples (Figure 5D). Lastly, focal adhesion gene PIK3CB was significantly upregulated after 30 days of culture aboard the ISS (Figure 5E). Focal adhesion has been shown to regulate cell proliferation and migration [49]. If CPCs are to be used in a cell-based approach for cardiovascular repair, they would need to be activated for optimal performance, similar to what we see following spaceflight.

### 2.6. MicroRNA Analysis

**Figure 6:**
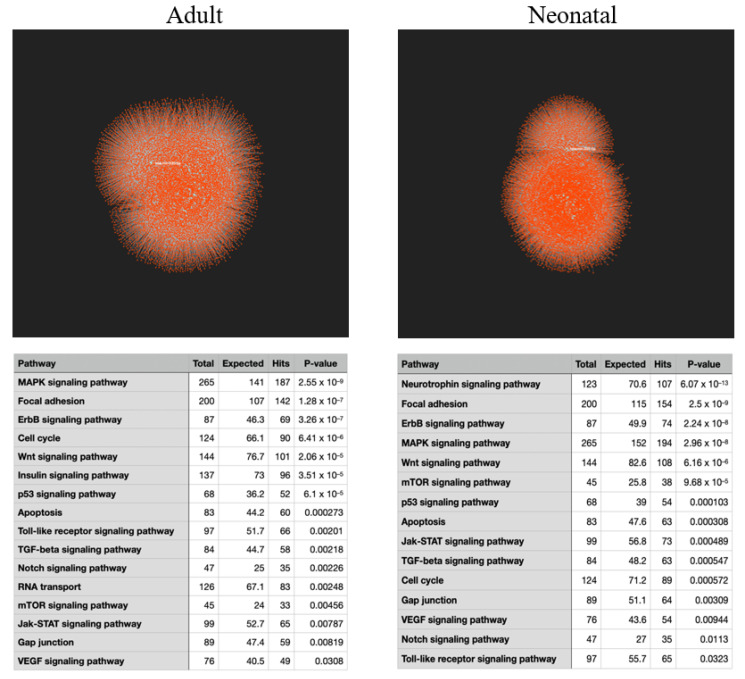
Predicted targets of miRNAs induced by spaceflight impact pathways related to cell cycle progression and survival. Differentially expressed miRNAs from flown adult and neonatal CPCs were mapped on a network using miRNet. KEGG analysis of predicted gene targets from adult and neonatal miRNAs reflects relevant regeneration and repair pathways.

In addition to whole transcript analysis, we investigated the effects of spaceflight on microRNA (miRNA) expression in adult and neonatal CPCs. A total of 89 differentially expressed miRNAs were significant in the flown adult CPCs (72 upregulated, 17 downregulated) and 124 differentially expressed miRNAs in the flown neonatal CPCs (110 upregulated, 14 downregulated). Using miRNet, an online miRNA target prediction software, we uploaded the differentially expressed miRNAs for the flown adult and neonatal CPCs to create a network of predicted target genes (Figure 6). Next, we performed a KEGG analysis based on the predicted targets of the uploaded miRNAs from the adult and neonatal samples, shown in Figure 6. Interestingly, many of the predicted pathways based on the miRNAs were similar to the pathways that were induced based on total transcriptomic analysis. This observation is due, in part, to the expression of multifunctional miRNAs such as the miRNA-17–92 cluster. Upregulated members of the miRNA-17–92 cluster positively regulate cell cycle progression [50] and proliferation [51], increase Yap1 expression [52], and protect against oxidative stress [53]. In our study, spaceflight increased the expression of miRNA-17 by 3.05- and 2.94-fold change in adult and neonatal CPCs, respectively.

## 3. Discussion

In this study, we investigated the effects of spaceflight on the genetic profile of human adult and neonatal CPCs. This is one of the first studies, to our knowledge, to investigate the effects of spaceflight on early-stage human cardiovascular progenitor cell clones from a full transcriptome perspective. The findings of this study provide insight into the mechanisms that regulate the cellular response to spaceflight and microgravity.

Short-term culture aboard the ISS induces incremental and reversible effects that mirror transcriptomic changes indicative of dedifferentiation and enhanced stemness in adult and neonatal CPCs. Gene expression changes induced by spaceflight in early-stage cardiovascular progenitors result in a transcriptomic profile with enhanced similarity to the stemness signature of iPSCs as well as other stem cell-related genes. Dedifferentiation reintroduces the proliferative nature of a cell by activating markers of a stem cell phenotype that help drive cell cycle re-entry [54]. Applying the process of dedifferentiation in an organ-specific context may therefore be beneficial for regenerative medicine. For example, forced expression of dedifferentiation factors via viral vector transduction reprogrammed rodent cardiomyocytes towards a progenitor-like state and enhanced their proliferative capacity [55]. The reprogramming factors involved in dedifferentiation include Yamanaka factors Sox2 and Klf-4. Here, we show that viral vector-independent induction of dedifferentiation markers can be achieved by short-term spaceflight.

Another significant transcript associated with stemness is TERT which promotes cell proliferation and survival [56]. TERT expression decreases with age under normal circumstances [57,58]; however, in our data, as well as in the NASA Twin Study, TERT expression is significantly elevated by spaceflight. TERT is also related to aging due to its role in inhibiting cellular senescence. As we initially expected to find that spaceflight would enhance senescence rather than promote stemness, we utilized several databases to compare the transcriptomic data in our study with well-documented signatures of senescence. Surprisingly, many of the relatively few senescence-inducing transcripts that were elevated in our data have dual roles in promoting cell cycle re-entry and survival. For example, the expression of megakaryocyte-associated tyrosine kinase (MATK) has been shown to positively regulate cell proliferation and survival across several in vitro models [59]. Similarly, Pim1 expression promotes cell proliferation and is involved in cardiac lineage commitment [60,61]. An alternative explanation of the expression of both senescence- and stemness-associated factors is a phenomenon referred to as the senescence-associated secretory phenotype (SASP) [62], where senescent-like cells secrete and promote the expression of stemness markers [63]. In vivo experiments have demonstrated that the SASP increases the expression of stemness markers in mouse keratinocytes and improves their regenerative capacity [64]. In early human CPCs cultured in space for 30 days, the molecular basis for the simultaneous induction of the group of genes identified here will require further investigation.

The Notch, ERBB, Wnt, and Hippo signaling pathways were identified as notably impacted by spaceflight based on gene expression analysis using IPA and DAVID. The transient activation of Yap1 as a consequence of the effect of the spaceflight environment on Hippo signaling benefits cell survival, proliferation, and regeneration as we have previously reported [36]. Further investigation into the effect of spaceflight on the whole transcriptome of CPCs revealed further insight into the mechanism of Yap1 induction. For example, GPCR signaling, which was significantly activated following culture aboard the ISS in early-stage CPCs, is capable of regulating the Hippo pathway via Rho GTPase inhibition of LATS1/2: the immediate upstream inhibitors of Yap1 [65]. Elevated Yap1 signaling directly impacts other pathways including ERBB and Notch due to activation of transcripts downstream of intranuclear Yap1 expression. Notch signaling normally decreases in cardiomyocytes with age, contributing to the decline in proliferation, but was elevated following culture aboard the ISS. Increased Notch activity in mouse cardiomyocytes stimulates proliferation and cell cycle re-entry while promoting anti-apoptosis gene expression [66]. Differentiation and heart development are regulated by crosstalk between Wnt and ERBB signaling pathways, both of which are induced by short-term spaceflight. Non-canonical Wnt is capable of activating ERBB4, resulting in enhanced cardiac commitment and cardiomyocyte formation from pluripotent stem cells [67]. Components of the ERBB signaling pathway, particularly neuregulins 1–4, are necessary for functional cardiovascular development, and emerging evidence suggests that activation of this pathway supports cardiomyocyte survival and cardiac function [68]. Focal adhesion formation is dependent on ERBB1, providing evidence that ERBB signaling is necessary for cellular adaptation to a foreign environment, such as the ISS environment [69]. Oxidative stress is attenuated by Yap1 and its downstream target SOD2, which prevents excessive accumulation of reactive oxygen species after injury [70,71]. Activation of these processes and pathways serves as a vital step in conditioning CPCs for survival in space, as demonstrated by the high viability (>90%) of these cells when returned live to Earth. Spaceflight induces several pathways and processes that are regulated by miRNAs whose expression was found to be coordinately regulated, as determined by our transcriptomic data.

Profiling miRNA expression during spaceflight has identified several miRNAs that have key regulatory roles in the cellular response to space [72]. In a recent study, Malkani et al. exposed mice to spaceflight-associated conditions to evaluate the expression of known miRNAs. From there, the top target genes were predicted and analyzed to determine the signaling pathways that were most likely to be induced by spaceflight. These included TGF-β signaling. Similarly, a group evaluating the effect of simulated microgravity on human umbilical cord endothelial cells found that microgravity altered the expression of miRNAs involved in cell proliferation, cell cycle regulation, TGF-β signaling, VEGF signaling, MAPK signaling, Jak-STAT signaling, focal adhesion, and wound healing [73]. In the present study, the predicted signaling pathway targets of induced miRNAs in CPCs cultured aboard the ISS for 30 days agree with the above-mentioned studies, suggesting a general effect of spaceflight on specific miRNAs and signaling pathways.

The cell type studied in our model functions in the context of cardiovascular repair on Earth when cultured under normal gravity conditions. Although current approaches to enhance cardiovascular repair have shown some benefit, there is significant room for improvement. Interestingly and unexpectedly, the above-mentioned transcripts and signaling pathways most impacted by spaceflight have critical roles in this process. Benefits of ERBB signaling, for example, have been applied in recent therapies for heart failure patients, where a phase I clinical trial is testing an NRG isoform associated with the ERBB signaling pathway as a therapy for heart failure [74]. Spaceflight induces NRG signaling in CPCs. In laboratory-based studies, the evidence for enhanced regeneration associated with Yap1 expression is well established in multiple small and large animal models. Notch signaling sustains cardiomyocyte proliferation and protects the heart from damage by limiting fibrotic scar tissue formation [75], and Wnt/β-catenin signaling is known to play a role in mediating the post-myocardial infarction response [76]. The concept that spaceflight has the potential to prime early-stage CPCs towards a regenerative cell type via transient and reversible dedifferentiation is unexpected but supported by the data which suggest that short-term culture aboard the ISS has the potential to enhance survival and select stemness properties. The genetic alterations discussed in the present study may be achievable via simulated microgravity on Earth in a shorter timeframe, as suggested by Fuentes et al. [11]. Transcriptomic analysis of simulated microgravity-induced changes remains to be investigated.

Understanding the influence of the spaceflight environment on humans and on specific cell types will require continued investigation. Transcriptomic analysis of the effects of spaceflight on human early-stage cardiovascular progenitor cells has contributed new information that may broaden the potential applications of cell culture in space. The differentially expressed transcripts reported here play significant roles in regeneration, survival, and proliferation and raise the possibility that short-term culture in space may benefit our life on Earth by activating signature pathways of stemness and survival in select cell types.

## 4. Materials and Methods

### 4.1. CPCs and Culture Aboard the ISS

Adult and neonatal CPCs were derived from discarded human cardiovascular tissue as previously described by our lab [77]. The Institutional Review Board at Loma Linda University approved the harvesting of cells from human patients following clinically necessary cardiovascular surgery. Collected tissue was digested with collagenase (Roche, Indianapolis, IN, USA) and passed through a cell strainer. Clonal expansion was performed and the expression of islet-1, MESP1, SSEA-1, and low levels of c-Kit were verified. Markers indicative of early-stage progenitor cells were identified in these cells.

Cell clones were cultured at the Kennedy Space Center ahead of launch in order to ensure optimal viability prior to integration (viability was 97.3–99.6%). In preparation for launch, cells were seeded into Biocell hardware (Bioserve, Boulder, CO, USA) at a density of 5000 cells per Biocell and loaded into plate habitats provided by Bioserve to maintain the cells at 37 °C and allow for gas exchange during launch. Live cells were launched aboard SpaceX CRS-11 and flown to the ISS. Aboard the ISS, plate habitats were transferred to the Space Automated Bioproduct Lab (SABL) which maintains the cells at 37 °C and 5% CO_2_. Cell culture media were changed on days 3, 8, 12, 16, 20, 24, 27, and 30. On day 30 after feeding, the plate habitats were transferred from SABL and packed in NASA-provided stowage for descent to Earth at 37°C. On day 31, upon retrieval, cell counts were performed and viability was assessed (viability was greater than 93%). Spaceflight enhanced the proliferative potential of adult and neonatal CPCs in this experiment [35]. The samples used for transcriptomics consist of three unique adult and three unique neonatal biological replicates. Upon retrieval, the cells were immediately placed in RNAProtect (Qiagen, Valencia, CA, USA) in order to maintain their genetic profile. Clonally identical adult and neonatal cells were cultured on Earth under normal gravity conditions as controls. RNA was purified using an RNeasy Mini Kit (Qiagen, Valencia, CA, USA) following the manufacturer’s protocol. RNA yield and quality were determined via RNA integrity number (RIN) before transcriptomic analysis.

### 4.2. RNA Sequencing

RNA samples were sent to the PrimBio Research Institute for transcriptome analysis (Exton, PA, USA). First, rRNA was removed from the total RNA sample using an rRNA removal kit from Illumina (San Diego, CA, USA) (cat# MRZG12324). Sequencing libraries were assembled with an Ion Total RNA-Seq Kit v2 from Thermo Fisher (Waltham, MA, USA) (Cat# 4479789). Nucleic acid binding beads from Ambion (Austin, TX, USA) were used to purify the cDNA library (Cat# 4479681) prior to PCR amplification. Agilent dsDNA High Sensitivity kit was used to determine the quality of the library (Agilent, Santa Clara, CA, USA). The samples were enriched via an Ion OneTouch ES instrument and an Ion PI™ Hi-Q™ OT2 200 Kit (Thermo Fisher, Waltham, MA, USA). Sequencing was performed using an Ion Proton sequencer (Thermo Fisher, Waltham, MA, USA) and a species-specific protocol for our samples. Next, sequence files were aligned to the human genome and quality was determined using the Strand NGS program. Normalization and quantification of the aligned reads were performed using the DEseq algorithm within the Strand NGS program. The Audic–Claverie test and the Benjamini–Hochberg correction test were used for statistical analysis. Significance was determined using a 2.0-fold change minimum cutoff.

### 4.3. Transcriptomic Analysis

Gene transcripts were sorted according to fold change, as previously mentioned, and uploaded to IPA (Qiagen, Valencia, CA, USA). Core analysis was performed with a specific parameter set for human cells. Categories specific to cancer and viral disease were removed and only categories with *p*-values > 0.05 were reported. Analysis through Database for Annotation, Visualization and Integrated Discovery (DAVID) was used to visualize pathways impacted by spaceflight-induced transcripts [78,79]. The lists of significantly induced gene transcripts from adult and neonatal age groups were uploaded to StemChecker (http://stemchecker.sysbiolab.eu, accessed on 29 March 2021) to determine stemness in flown CPCs. StemChecker refines the uploaded lists by identifying genes that overlap with published literature in which stem cell maintenance or stemness signatures are reported. Our uploaded gene sets were compared to the expression profile of iPSCs.

The database of senescence-associated genes was downloaded from the CellAge website (https://genomics.senescence.info/cells/, accessed on 29 March 2021). From here, the genes specifically labeled as pertaining to cancer types were excluded from our analysis. Differentially expressed miRNAs from adult and neonatal CPC datasets were uploaded to DIANA mirPath v.3 [80] (http://snf-515788.vm.okeanos.grnet.gr/, accessed on 29 March 2021) and miRNet (https://www.mirnet.ca, accessed on 29 March 2021). Target gene networks were automatically generated by miRNet. KEGG analysis was performed based on predicted targets of the uploaded miRNAs.

## Data Availability

The data presented in this study are openly available in FigShare and are accessible through 10.6084/m9.figshare.14125742. Additional data can be made available upon reasonable request to the corresponding author.

## Abbreviations

CPC	Cardiovascular progenitor cell
DAVID	Database for Annotation, Visualization and Integrated Discovery
EMT	Epithelial–mesenchymal transition
ESC	Embryonic stem cell
GPCR	G protein-coupled receptor
iPSC	Induced pluripotent stem cell
IPA	Ingenuity Pathway Analysis
ISS	International Space Station
KEGG	Kyoto Encyclopedia of Genes and Genomes
NASA	National Aeronautics and Space Agency
NRG	Neuregulin
